# High-Resolution 3D Heart Models of Cardiomyocyte Subpopulations in Cleared Murine Heart

**DOI:** 10.3389/fphys.2022.779514

**Published:** 2022-05-18

**Authors:** Huiying Ren, Zhaoli Pu, Tianyi Sun, Tangting Chen, Leiying Liu, Zhu Liu, Christopher O’Shea, Davor Pavlovic, Xiaoqiu Tan, Ming Lei

**Affiliations:** ^1^ Laboratory of Medical Electrophysiology, Ministry of Education, Collaborative Innovation Center for Prevention and Treatment of Cardiovascular Disease/Institute of Cardiovascular Research, Luzhou Medical College, Luzhou, China; ^2^ Department of Cardiology, The Affiliated Hospital of Southwest Medical University, Luzhou, China; ^3^ Department of Pharmacology, University of Oxford, Oxford, United Kingdom; ^4^ Institute of Cardiovascular Sciences, College of Medicine and Dental Sciences, University of Birmingham, Birmingham, United Kingdom

**Keywords:** HCN4 expression (HCN4+) pacemaker cells, Pnmt+ cell-derived cardiomyocytes (PdCMs), heart tissue-clearing, light-sheet fluorescence microscopy, 3D volume heart models, optogenetics

## Abstract

Biological tissues are naturally three-dimensional (3D) opaque structures, which poses a major challenge for the deep imaging of spatial distribution and localization of specific cell types in organs in biomedical research. Here we present a 3D heart imaging reconstruction approach by combining an improved heart tissue-clearing technique with high-resolution light-sheet fluorescence microscopy (LSFM). We have conducted a three-dimensional and multi-scale volumetric imaging of the ultra-thin planes of murine hearts for up to 2,000 images per heart in x-, y-, and z three directions. High-resolution 3D volume heart models were constructed in real-time by the Zeiss Zen program. By using such an approach, we investigated detailed three-dimensional spatial distributions of two specific cardiomyocyte populations including HCN4 expressing pacemaker cells and Pnmt^+^ cell-derived cardiomyocytes by using reporter mouse lines Hcn4^DreER/tdTomato^ and Pnmt^Cre/ChR2−tdTomato^. HCN4 is distributed throughout right atrial nodal regions (i.e., sinoatrial and atrioventricular nodes) and the superior-inferior vena cava axis, while Pnmt^+^ cell-derived cardiomyocytes show distinct ventral, left heart, and dorsal side distribution pattern. Our further electrophysiological analysis indicates that Pnmt + cell-derived cardiomyocytes rich left ventricular (LV) base is more susceptible to ventricular arrhythmia under adrenergic stress than left ventricular apex or right ventricle regions. Thus, our 3D heart imaging reconstruction approach provides a new solution for studying the geometrical, topological, and physiological characteristics of specific cell types in organs.

## Introduction

Biological tissues are naturally three-dimensional (3D) structures and are usually opaque. This causes a major problem in deep imaging for defining the spatial distribution and positioning of specific cell types in intact organs, especially in highly complex tissues such as the heart and the brain. While modern fluorescent microscopic platforms, such as wide-field fluorescent microscopy or confocal scanning microscopy, have a high spatial resolution for imaging cellular structures, they suffer from insufficient axial resolution, as well as phototoxic damage if prolonged imaging is required (Sanderson et al., 2014). An alternative approach is to combine the traditional histological sectioning approach to produce thin-sliced tissue specimens (e.g., immunohistological staining on sectioned tissue samples) with post-imaging 3D reconstruction through computation. Histological sectioning also has significant limitations including tissue structure distortion and displacement, destruction of cell connections, and low spatial resolution when slicing samples.

The recently emerged tissue-clearing approach provides an exciting opportunity to solve such problems by allowing efficient fluorescent labeling and rapid 3D volumetric imaging of intact tissues, organs, and even entire organisms (Susaki and Ueda, 2016; Ueda et al., 2020). After the clearance, the tissue becomes transparent, so the imaging depth possible is less attenuated by the tissue sample. Such deep tissue visibility enables the interrogation of whole organs and even whole organisms without the need for sectioning the target tissue (Ueda et al., 2020). Clearing also removes strongly light-scattering and light-absorbing components of tissue and equalizes the refractive index of the imaging medium to that of the tissue (Verveer et al., 2007). Currently, three major tissue-clearing methods are being used: hydrophobic- (‘solvent’), hydrophilic- (‘aqueous’), and hydrogel-based methods (Susaki et al., 2015; Susaki and Ueda, 2016; Tainaka et al., 2016; Ueda et al., 2020). The hydrophobic clearing approach uses organic solvents and results in quick transparency of the tissue (Vigouroux et al., 2017). The 3D imaging of solvent-cleared organs (3DISCO) is a good example developed by Ertürk and colleagues (Ertürk et al., 2011; Ertürk et al., 2012). It is able to clear a complete adult mouse brain within 2 days (Ertürk et al., 2011; Ertürk et al., 2012). The protocol is simple and straightforward, which only involves a series of incubation of the samples. 3DISCO is now widely applied in neuronal research, stem and cancer cells in rodent and human specimens (Ertürk et al., 2011; Ertürk et al., 2012; Acar et al., 2015; Garofalo et al., 2015; Oshimori et al., 2015; Tanaka et al., 2017; von Neubeck et al., 2018). The disadvantage of this method is that the pretreatment solution contains H_2_O_2_ and methanol, which damage and even remove most of the epitopes for antibodies staining. Thus, immunohistochemistry remains a challenge in using such an approach (Renier et al., 2016).

Compared to the hydrophobic clearing method, hydrophilic tissue clearing has the advantages of high biocompatibility, biosafety, and the integrity of protein function although the transparency is not as good as the hydrophobic clearing method (Ueda et al., 2020). During the clearing process, hydrogen bonds are formed to link the clearing reagents and tissue components as well as proteins and water molecules (Ueda et al., 2020). Therefore, it greatly protects the structure of specimens and fluorescent proteins. CUBIC is a typical representative. CUBIC is composed of a series of imaging cocktails including CUBIC-L (use for delipidation) and CUBIC-R (RI match). It is reported that the clearing performance of CUBIC is even better than the hydrophobic clearing methods. Together with the advantages of hydrophilic clearing methods, CUBIC has been applied and illustrates the 3D structures with immunohistochemistry with antibodies for the adult mouse brain, heart, lung, stomach, and intestine (Susaki et al., 2014; Tainaka et al., 2014; Susaki et al., 2015; Kubota et al., 2017; Tainaka et al., 2018).

The third method is the hydrogel-based tissue clearing method, its typical application is ‘cleared lipid-extracted acryl-hybridized rigid immunostaining/*in situ* hybridization-compatible tissue hydrogel’ (CLARITY) (Chung et al., 2013). Unlike the hydrophilic clearing method, it forms hydrogels by covalent links to uniformly remove the lipid with fewer structure damages and bimolecular losses (Chung et al., 2013). The clearing force is driven by electrophoresis and simple passive, which improves the clearing performance but is time-consuming compared to the other two methods (Ueda et al., 2020).

Despite a plethora of advanced cleaning methods, most of them are designated for brain and nervous tissues and lack the consideration of the nature of the heart (Kolesová et al., 2021). Unlike the brain, the heart is enriched in connective tissues and autofluorescence (Sands et al., 2022). The heart consists of clustered cardiomyocytes arrayed into ventricular transmural layers from subepicardial to subendocardial tissues (Matryba et al., 2019). Packed cardiomyocytes are surrounded by thicker connective tissue (perimysium) (Ho, 2009). The heme a precursor to hemoglobin within the myocardium is pigmented, leading to autofluorescence, which in turn impedes the penetration of light when visualization (Matryba et al., 2019). Researchers compared and optimized the protocols for clearing experiments on heart and vasculature tissue. DISCO is a successful trial in heart clearing. By combining 3DISCO and immunostaining, the vasculature development of the human heart was illustrated by showing the smooth muscle-specific alpha-actin (SMA) (Belle et al., 2017; Epah et al., 2018). The conduction system of the mouse heart was also reconstructed by applying iDISCO + tissue clearing method (Goodyer et al., 2019). However, the drawback of DISCO is the poor preservation of GFP fluorescence (Pan et al., 2016). CUBIC is commonly used for heart clearing research because it can effectively remove the autofluorescence and allow observation of the detailed structure of the whole heart (Susaki et al., 2015; Susaki and Ueda, 2016). Kolwsová and colleagues tested different clearing protocols for imaging whole embryos, embryonic, and adult hearts with GFP genetic fluorescence. The results pointed out that DBE (dibenzylether) did not preserve the GFP signals and triggers the shrinking of tissue; CLARITY improved the clearing effect but compromise the GFP signals; SCALE clearing led to good clearing until E12.5 but failed to function well in large-scaled samples in later stages; CUBIC showed better performance of clearing with imaging although adult hearts took a longer time (7–14 days) (Kolesová et al., 2021).

Recently, CUBIC clearing of rat hearts by perfusing CUBIC solutions through the coronary circulation system was also described by Sands et al. (2022). When tissue clearing combines with light-sheet fluorescence microscopy (LSFM), 3D imaging of biological samples with high speed and low photo-bleaching can be achieved. Hence, this approach has emerged as a powerful tool for biological research. LSFM itself provides higher speed, better signal-to-noise ratio, lower level of photo-bleaching, and improved optical penetration depth than conventional wide-field fluorescent microscopy or confocal microscopy (Tomer et al., 2013). Furthermore, it has the ability to selectively illuminate an ultra-thin plane of the sample via the application of a sheet of light orthogonal to the detection path (Tomer et al., 2013).

Recent work has identified a previously unknown subpopulation of cardiomyocytes (CMs): Pnmt^+^ cell-derived cardiomyocytes (PdCMs) and their unique left-side preferential distribution (Wang et al., 2017). We developed a novel optogenetic mouse strain by crossing Pnmt-Cre mice with Ai27D mice that expressed an improved channelrhodopsin-2(ChR2)/tdTomato fusion protein, following excision of a STOP sequence that was flanked with -loxp sequences. The fluorescence signal from tdTomato provided an excellent endogenous marker to identify PdCMs. Coronal sections of hearts agreed with earlier work that shows the left atrium, left ventricle, and interventricular septum were particularly rich in PdCMs (Wang et al., 2017). In remarkable proximity to earlier quantitative approaches, an average of 86% of ChR2/tdTomato + cells were on the left side of the heart. Moreover, this study now quantitatively reported the contribution of PdCMs to the overall number of CMs; PdCMs estimates suggest that approximately 50% of the left atrium, and 21% of the left ventricle CMs are PdCMs—in agreement with the extensive distribution of Pnmt-derived cardiomyocytes in the left atrium in previous studies (Ebert et al., 1996). These estimates provide concrete quantitative evidence of the significance of PdCMs in terms of development heterogeneity in the heart (Wang et al., 2017). Moreover, in agreement with previous studies, we reported a great deal of co-localization of HCN4+ cells with tdTomato + cells in the AVN, but with less so in the SAN—suggesting that the AVN has a richer population of PdCMs than the SAN (Wang et al., 2017; Ni et al., 2017).

In this study, we present a new 3D heart imaging reconstruction approach to generate murine heart volume models of spatial distribution of specific cell types (e.g., Pnmt-Derived Cardiomyocytes) obtained by combining an improved heart tissue clearing method with high-resolution LSFM. The experiment has three major steps. First, the heart sample is perfused by physiological solution in the Langendorff perfusion system allowing the effective clearance of blood and tissue clearing. Such a step is critical for reducing cardiac tissue autofluorescence background. Second, the heart then undergoes a number of methodological interventions enabling efficient tissue clearing. Crucially, we found a slow perfusion rate for clearing solution for 24–48 h *via* coronary circulation system provides the best clearing protocol for obtaining a transparent heart. Third, a high-resolution LSFM is used for imaging transparent hearts. Finally, high-resolution 3D volume heart models are constructed in real-time by the Zeiss Zen program.

Therefore, we successfully developed a series of 3D volume models to define the spatial distributions of specific cardiomyocyte populations, which is comparable to the computational 3D models based on the histological images. Our approach is efficient, not necessitating tissue sectioning, maintaining optimal tissue structure and organization, and has a much higher axial and spatial resolution.

## Technology Development

### Animal Models

The Pnmt^Cre/ChR2−tdTomato^ mouse line was generated by crossing Pnmt-Cre mice with B6. Cg-*Gt(ROSA)26Sor* ^*tm27.1(CAG-COP4H134R/tdTomato)Hze*^/J strain (Stock No. 012567, Jackson Labs) as we described previously (Wang et al., 2017).

The second mouse line HCN4^Dre/tdTomato^ was generated by crossing C57BL/6-Hcn4^em1(kozak−DreER-WPRE-A)/Smoc^ mice with C57BL/6-Igs2^em1(CAG−RSR-tdTomato)/Smoc^ mice from Shanghai Model Organisms Center, Inc. (Shanghai, China). The model generation was performed with the approval of the Animal Care and Use Committee of the Southwest Medical University (Sichuan, China) (No: 20160930) in conformity with the NIH Guide for the Care and Use of Laboratory Animals.

## Sample Preparation and Tissue Clearing

Step 1 Heart preparation

**FIGURE 1:**
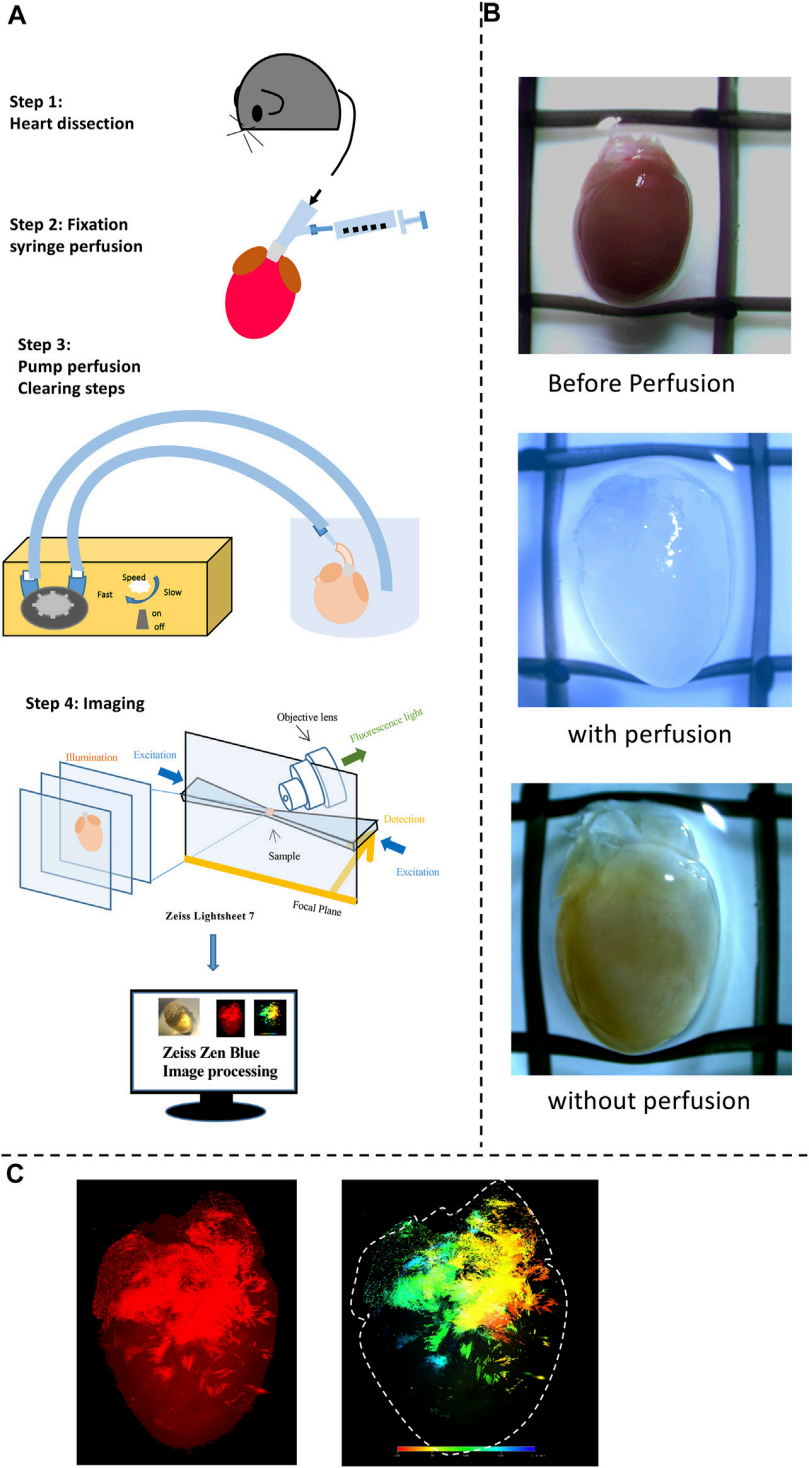
A workflow for tissue clearing and examples of 3D reconstruction. **(A)** A schematic overview of the workflow of the study and the process of the experimental procedures. Heart dissection was conducted. Afterward tissue fixation and clearing were done to obtain transparent hearts. Finally, Fluorescence images for detecting fluorescent positive cells were obtained using Zeiss Fluorescent microscopy (provide the model details here) in transparent hearts; Diagram illustrating the procedure for the 3D reconstruction achieved using the ZEISS ZEN software to present both Tdtomato fluorescence and depth color code models. 3D volume data (Videos 1–4) was visualized in Paraview following Gaussian smoothing and down-sampling. **(B)** Images of Heart samples showing the appearance before, with, and without the perfusion. **(C)** Examples of fluorescence and depth code images for final products.

The general procedure has been demonstrated in Figure 1A. All mice were killed via cervical dislocation in accordance with the Animals Scientific Procedures Act (1986). Before killing, the mice were heparinized by intraperitoneal injection of heparin sodium physiological salt solution (Sigma-Aldrich, 500 U/mL) 0.2 ml. The heart was excised, cannulated, and mounted onto a Langendorff system, then perfused (ﬂow rate: 3 ml/min; Watson-Marlow Bredel Peristaltic pumps, model 505S, Falmouth, Cornwall, UK) with Krebs’ Ringer (KR) (mM) (NaCl: 119, NaHCO_3_: 25, Glucose: 10, Na Pyruvate: 2, KCl: 4, MgCl_2_: 1, KH_2_PO4: 1.2, and CaCl_2_: 1.8) warmed solution (37°C) for 5–10 min. The heart was removed from the Langendorff system and then flushed with phosphate-buffered saline (PBS) briefly. Afterwards, the heart was continued prepared by steps 2 and 3 (Figure 1A).

Step 2 Fixation

Followed by 4% paraformaldehyde (PFA) in phosphate buffer solution for fixation for 2 h at 4 °C. After a 15 ml PBS wash, the whole heart was then immediately perfused with CUBIC-P for 30 min, which is a chemical allowing efficient perfusion-fixation compatibility with downstream steps. This CUBIC-P is specially designed for organs with cavity structures such as hearts.

Step 3 Perfused Clearing

After washing out the remaining PFA and CUBIC-P with PBS, the heart was treated with a lipid-removal chemical named CUBIC-L for delipidation. This process was conducted at 37°C for 5°days. Perfusion is crucial at this stage to convey the reagent, enabling a better clearing. Because the mouse models we used have genetically-encoded fluorescence labeling of the cells of interest, no staining is required. Followed by washing, the heart was then treated with 50% CUBIC-R diluted by distilled water 1 day and then 100% CUBIC-R reagent to adjust the refractive index of the organs for at least 5days.

Step 4 Light-sheet Imaging

The transparent heart was illuminated by Zeiss 7 LSFM. The adult murine heart (8 weeks old) is approximately 1.3°cm × 0.7cm x 0.7 cm in height, width, and thickness. This LSFM with dedicated optics, enhanced sample chambers, and designated sample holders enables high-quality whole field imaging of the murine heart. LSFM separates the fluorescence of excitation and detection into two solo light paths. In this case, the axis of illumination is perpendicular to the detection axis as shown in Figure 1A, allowing the illumination of a single thin section of the heart at one point and thus generating inherent sections from the focus plane by merely exciting the fluorescence (Matsumoto et al., 2019). As a result, no pinhole or image processing procedures are needed. Unlike normal confocal fluorescent microscopes, LSFM is able to collect the light from the focus plane on the pixels rather than pixel by pixel, infinitely improving the resolution for the subsequent 3D reconstruction. After imaging, the features and details of the heart were captured and reconstructed for the 3D model by using Zeiss Zen Blue software. The stitching program was processed to combine multiple images with overlapped fields of view to generate high-resolution images. Fluorescent mode and depth code mode was selected to demonstrate the distribution of cell subpopulations of interest at three-dimensional presentation. A series of image galleries were collected at certain intervals of layers to show the distribution at a two-dimensional presentation. The raw video was recorded directly after the illumination without using other processing software. The thickness for PdCM and HCN4 hearts are 6.113 and 8.352 mm respectively. The total magnification was 4.22. The emission filter bandpass was 575–615 nm. High-resolution 3D volume heart models are constructed in real-time by the Zeiss Zen program. 3D iterative deconvolution was applied. By applying the maximum intensity, the reconstruction of the whole cardiac architecture was achieved to present clearly Tdtomato fluorescence by contrast with the background area in the transparent heart. The depth color code was employed to detect the depth of the signals which provided the accurate location of fluorescence in both epicardium and endocardium indicated by the color spectrum.

## 3D Data Construction

The genetic fluorescence of the mouse models was captured by the Zeiss Light Sheet fluorescence microscope Lightsheet 7. After stitching from raw files, 3D movies were recorded and further processed in Zen Blue software. On the 3D View module, the maximum intensity was adjusted to reduce the background and illuminate the fluorescent positive cells throughout the whole heart (online Video 1, 3). The depth color code was applied to show the specific location of the signals (online Video 2, 4). Visualizations were created by rotating the 3D volume and adding keyframes to the record module.

## Electrocardiography (ECG) Recording With programmed Light Stimulation (PLS) and Programmed Electrical Stimulation (PES)

Optical stimulation of ChR2 light-sensitive channel. Whole hearts, tissues, or single cells were paced through the activation of ChR2 light-sensitive channels. This was achieved by the delivery of 470 nm blue light pulses (10 ms pulse width) generated by OptoFlash (Cairn Research, UK). Pulses were triggered by a 1,401 digitizer and Spike 2 software (Cambridge Electronic Design, UK). Approximate blue light intensity was measured with an 818-ST2 Wand Detector connected to an 843 R Power meter (both Newport Corporation, CA, United States ) and expressed normalized for the area being illuminated through simulating the average light intensity reached to the surface of the tissue by mimicking the distance of fiber-heart.

Langendorff-perfused *ex vivo* hearts from Pnmt^Cre/ChR2^ mice were subjected to PLS or PES while ECG was being measured from an electrode placed on the left ventricle of the heart. Two protocols were carried out as follows: 1) continuous pacing protocol: Stimuli were delivered continuously with a constant frequency. 2) S1S2 pacing protocol: a pacing train of eight stimuli (S1) was delivered at a basic cycle length of 100 ms, with a single (S2) premature extra stimulus introduced at progressively shorter intervals until arrhythmia was induced or the ventricular refractory period was reached. (C) burst pacing protocol: multiple burst stimuli with progressively reduced stimulus intervals (from 80 to 30 ms) were delivered. Ventricular tachycardia was defined as six or more consecutive premature ventricular waveforms; tachycardia with regular waveforms defined as VT and VF was characterized by irregular fibrillating waveforms.

## Results and Discussion

**FIGURE 2:**
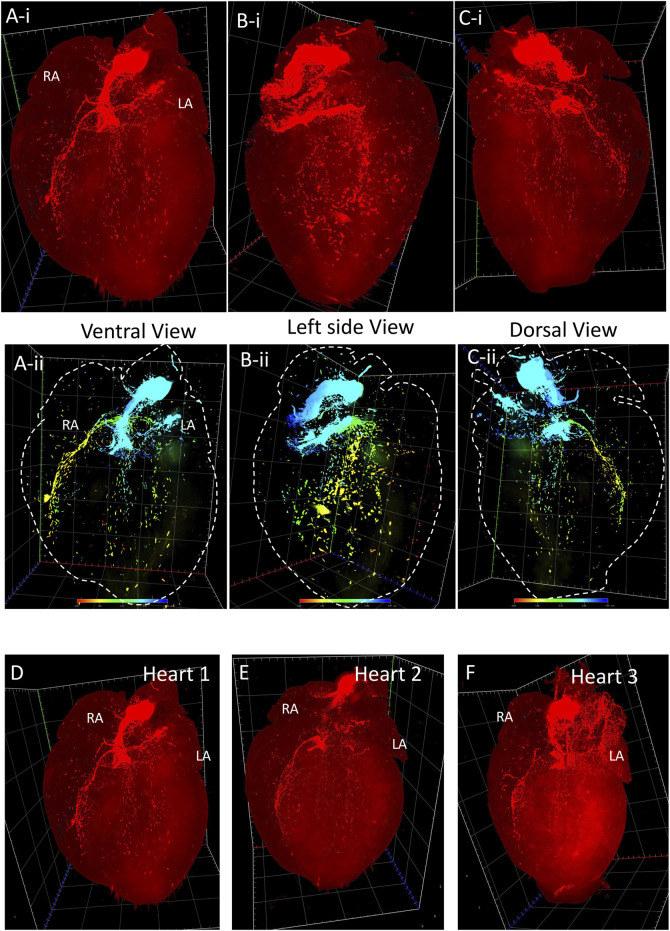
3D volume reconstruction of the spatial distribution of HCN4+ pacemaker cells in adult Hcn4^DreER/tdTomato^ mouse heart. **(Ai–Ci)** The tdTomato fluorescence images show the distribution of HCN4+ cells from the ventral, left side, and dorsal view respectively (also see online video 1, heart size: 10.05 mm × 12.01 mm x 7.565 mm). **(Aii–Cii)** The depth code image showing the distribution of HCN4+ cells from the ventral, left side, and dorsal view respectively, corresponding to **(Ai–Ci)** (also see online video 2). **(D–F)** Three representatives of tdTomato fluorescence image showing the consistent signal pattern in adult Hcn4^DreER/tdTomato^ mouse hearts. (n number = 3). LA: left atrium; RA: right atrium.

**FIGURE 3:**
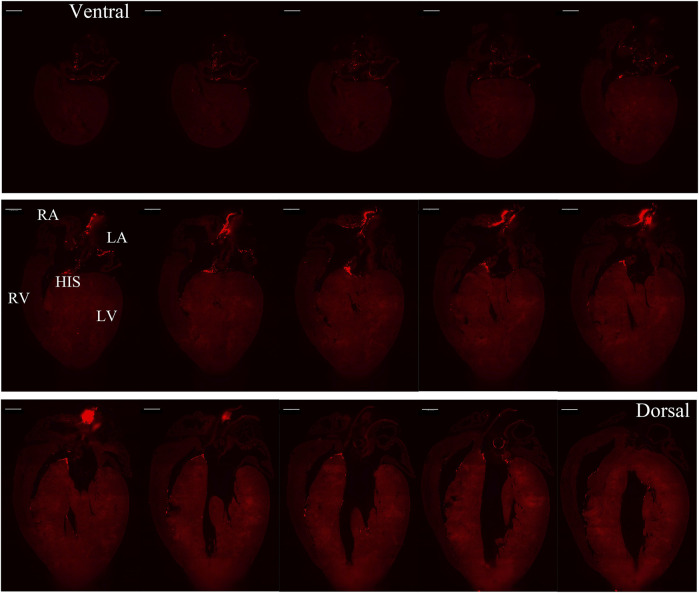
Selected representative coronal sections of Hcn4^DreER/tdTomato^ showing the distribution of HCN4+ cells from ventral side to dorsal side in adult mouse heart. Scale bar: 1,000 μm.

Figure 1 illustrates a schematic overview of the study design and experimental procedure. We first determined the spatial distribution of cardiac pacemaker cells by using potassium/sodium hyperpolarization-activated cyclic nucleotide-gated channel 4 (HCN4) as a marker. Figure 2 presents the 3D volume reconstruction of the spatial distribution of HCN4 positive pacemaker cells in the heart (also shown in online videos 1 and 2). HCN4 is distributed throughout the right atrium with heavy staining localized in the right atrial nodal regions (i.e., sinoatrial and atrioventricular nodes). The distribution of the HCN4 along the superior-inferior vena cava axis is in line with recent observations by Brennan et al. (2020) and supports the wandering pacemaker hypothesis with two competing right atrial pacemakers localized near the superior vena cava and the inferior vena cava. Selected images captured using LSFM imaging representing coronal sections from an adult tdTomato mouse heart show the distribution of HCN4^+^ pacemaker cells in adult HCN4^Dre/tdTomato^ mice from ventral to dorsal, allowing visualizing the regional distribution of HCN4 positive pacemaker cells in two dimensions (Figure 3). High-resolution images are available in online videos 1 and 2. The concurrent use of a programmed high-resolution LSFM imaging allowed for the capture of ∼2,000 ultra-thin plane images from a single murine heart with a spatial resolution of 1.54 µm × 1.54 µm × 4.01 µm per pixel respectively in the x-, y- and *z*-direction, in corresponding to 5,113 × 7,325 × 1,254 pixels in scaled image size.

**FIGURE 4:**
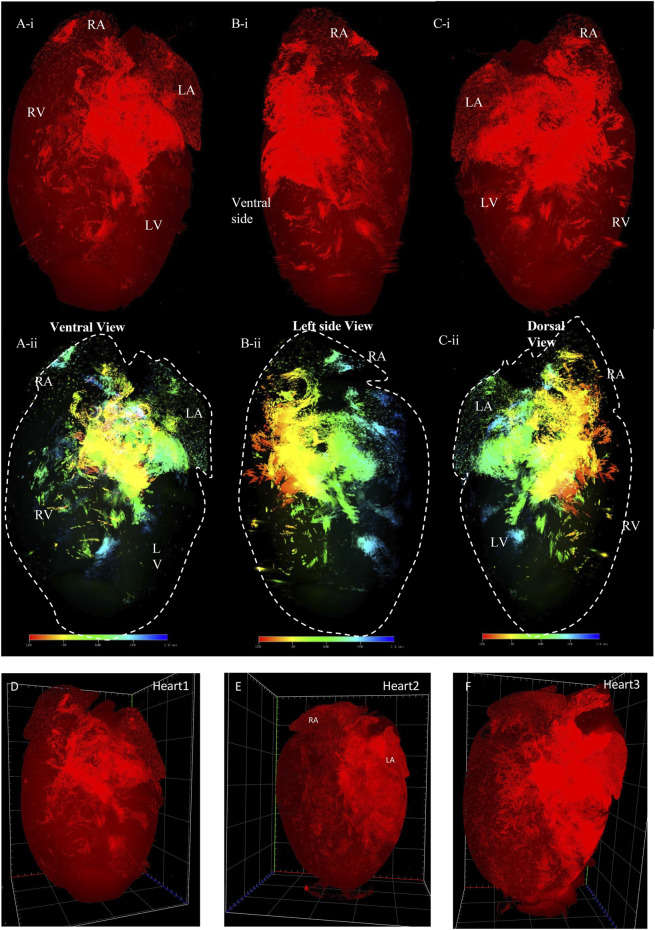
3D volume reconstruction of the spatial distribution of Pnmt + cell-derived cardiomyocytes (PdCMs) in adult Pnmt^Cre/ChR2−tdTomato^ heart. **(Ai–Ci)** The tdTomato fluorescence images show the distribution of PdCMs from the ventral, left side, and dorsal view respectively (also see online video 3, heart size: 7.86 mm × 11.26 mm x 6.113 mm). **(Aii–Cii)** The depth code image showing the distribution of PdCMs from the ventral, left side, and dorsal view respectively, corresponding to **(Ai−Ci)** (also see online video 4). **(D–F)** Three representatives of tdTomato fluorescence image showing the consistent signal pattern in adult Pnmt^Cre/ChR2−tdTomato^ mouse hearts. (n number = 3). LA: left atrium; RA: right atrium. LV: left ventricular; RV: right ventricular.

In the second series of models, we determined the spatial distribution of Pnmt^+^ cell-derived cardiomyocytes (PdCMs) that we discovered recently (Wang et al., 2017). Figure 4 presents examples of two 3D volume reconstruction models of the spatial distribution of Pnmt^+^ cell-derived cardiomyocytes (PdCMs) in adult Pnmt^Cre/ChR2−tdTomato^ hearts (also shown in the online videos 3 and 4). The use of the same programmed high-resolution LSFM imaging for the capture of ∼2,000 ultra-thin plane images from a single murine heart with similar spatial resolution in the x-, y-, and *z*-direction as obtained in HCN4^Dre/tdTomato^ hearts. The 3D volume model illustrates the reconstruction of the spatial distribution of Pnmt^+^ cell-derived cardiomyocytes (PdCMs) in adult Pnmt^Cre/ChR2−tdTomato^ heart. The tdTomato fluorescence is present from ventral, left, and dorsal views, respectively (Figures 4A–C). We also use depth code showing the localization of PdCMs in the heart from the ventral, left side, and dorsal side, respectively (Figures 4D–F). The localization of PdCMs has not been shown previously in a computational 3D representation with such a high spatial resolution. In Figure 4, the multiple views are given and detailed structure and localization, and depth of the PdCMs are shown.

**FIGURE 5:**
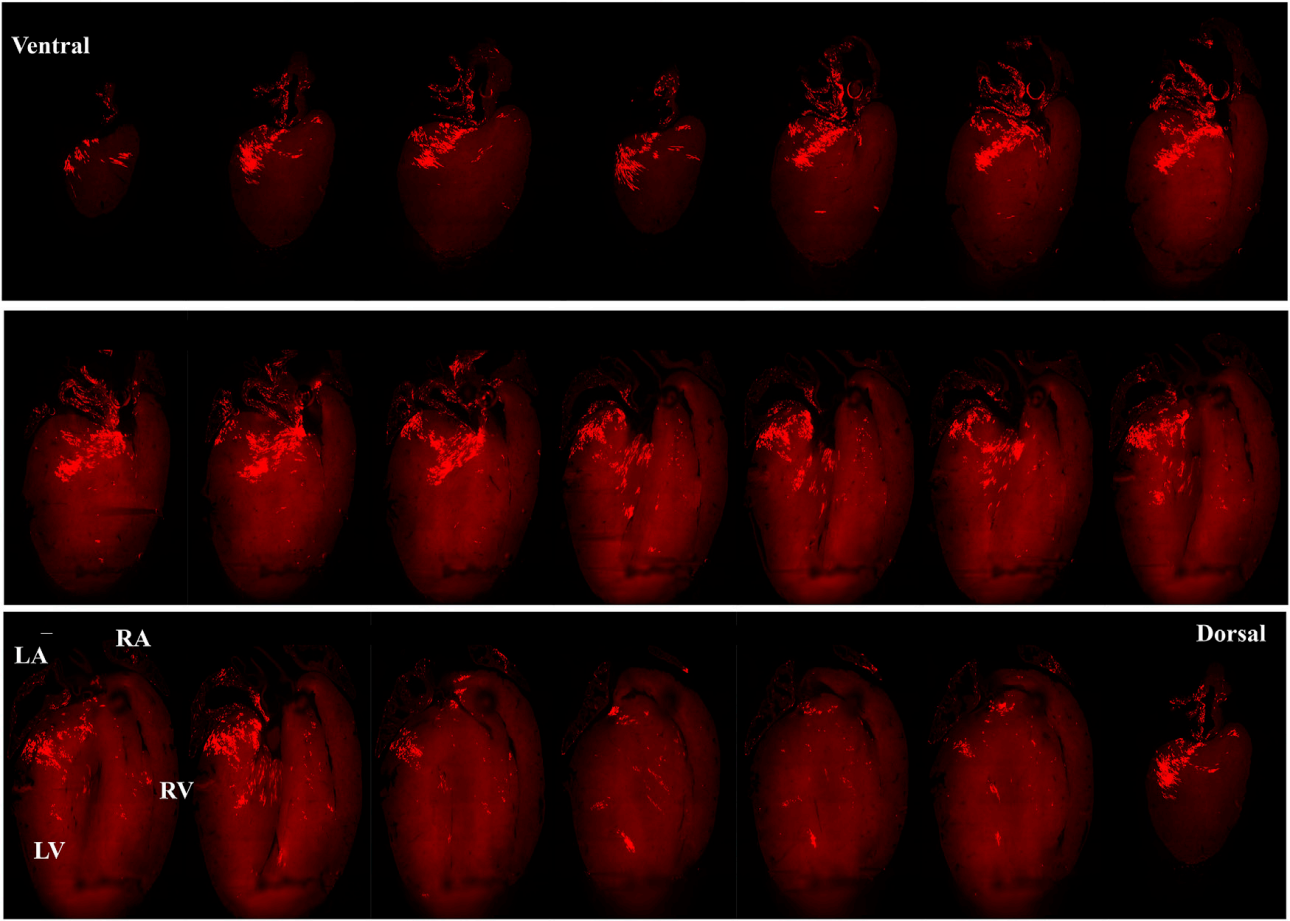
Selected representative coronal sections from an adult Pnmt^Cre/ChR2−tdTomato^ mouse heart showing the distribution of PdCMs from ventral side to dorsal side. Scale bar: 1,000 μm.

Figure 5 presents images captured by LSFM imaging representing coronal sections from an adult ChR2/tdTomato mouse heart. This shows the distribution of PdCMs from the ventral side to the dorsal side, allowing visualizing of the regional distribution of PdCMs in a two-dimensional manner.

**FIGURE 6:**
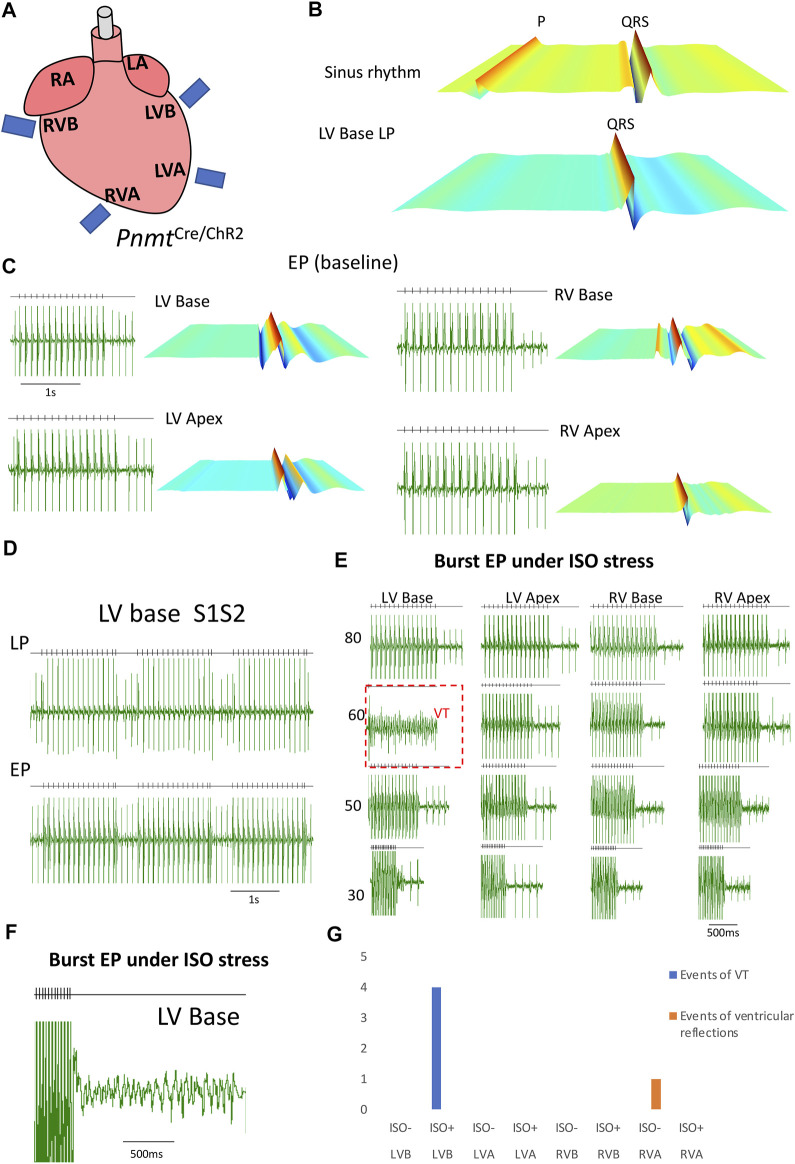
Controlling heart rhythm with selective optogenetic stimulation and electrical pacing under *β*-adrenergic stress conditions induced by administration of isoprenaline (ISO) compared with a baseline condition in an adult Pnmt^Cre/ChR2m^ mouse model. **(A)**. Localized light pacing and electrical pacing in different regions of the heart. **(B)**. The upper panel shows a representative ECG recording of a Pnmt^Cre/ChR2^ heart in intrinsic sinus rhythm. The bottom panels show representative ECG recordings of this heart paced by targeting blue light pulses to LV base regions respectively. “P” in representative ECG recordings stands for “P wave”. **(C)**. Examples of ECG recordings in the four different regions of Pnmt^Cre/ChR2^ mouse hearts under baseline conditions. **(D)**. ECG was recorded in the LV base of a Pnmt^Cre/ChR2^ heart by programmed light stimulation (PLS) and electrical pacing with the S1S2 protocol. **(E)**. ECG of Pnmt^Cre/ChR2^ in four different regions with frequency-dependent pacing under *β*-adrenergic (ISO) stress conditions. **(F)**. A typical example of VT occurred by burst electrical pacing under *β*-adrenergic stress conditions. **(G)**. Summary of the occurrence of VT in four different regions with frequency-dependent pacing under baseline and *β*-adrenergic stress conditions. The left ventricular base of hearts showed higher occurrences of VT under *β*-adrenergic stress. (n number = 5). LA: left atrium; RA: right atrium. LV: left ventricular; RV: right ventricular; LVB: left ventricular base; LVA: left ventricular apex; RVB: right ventricular base; RVA: right ventricular apex.

Figure 6 describes controlling heart rhythm with regional selective stimulation by electrical pacing under *β*-adrenergic stress conditions. First, by applying localized light pacing, we determined the light pacing responding region corresponding to the localization of Pnmt-derived cardiomyocytes (Figure 6A). In the ventricle, among four divided regions tested, only the left ventricular base responded (Figure 6B). We further conducted electrophysiological analysis compared ECG of PnmtCre/ChR2 in four different regions with frequency-dependent pacing under *β*-adrenergic (ISO) stress conditions. As shown in Figures 6E–G, the left ventricular base of hearts showed higher occurrences of VT under *β*-adrenergic stress.

**FIGURE 7:**
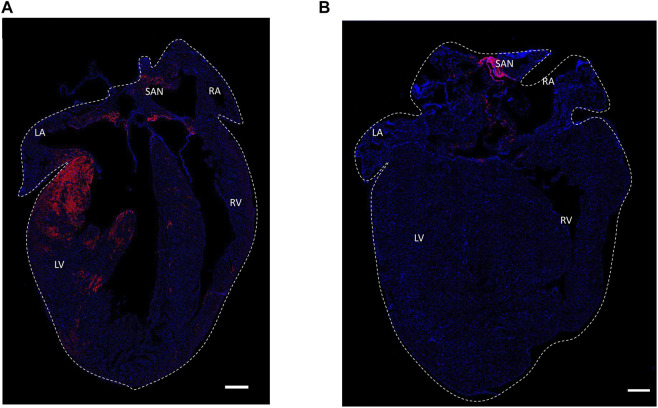
Histological section confocal microscopic images of expression of distribution patterns of the HCN4^+^ cells in Hcn4^DreER/tdTomato^ heart **(A)** and Pnmt^+^/Tdtomato positive cells in Pnmt^Cre/ChR2−tdTomato^ heart **(B)**. Tdtomato fluorescence signal indicating PdCMs was observed on the left side of the heart and SAN region. **(B)** Tdtomato fluorescence signal was mainly observed in the SAN and AVN regions in Hcn4^DreER/tdTomato^ heart. Blue: nuclear staining by DAPI. Scale bar: 500 μm. Online video 1: The video was generated to show the HCN4+ cells (tdTomato) in an adult mouse heart. The video can be found in the following link: https://figshare.com/s/26bbc6c38ae7a01c7778heart size: 10.05 mm × 12.01 mm x 7.565 mm. Online video 2: The video was generated to show the depth details of HCN4+ cells in the adult mouse heart. The video can be found in the following link: https://figshare.com/s/21c472847c46f0936dddheart size: 10.05 mm × 12.01 mm x 7.565 mm. Online video 3: The video was processed to show the PdCMs (tdTomato) in the adult mouse heart. The video can be found in the following link: https://figshare.com/s/01471ea1c3eda4f6bc43.heart size: 7.86 mm × 11.26 mm x 6.113 mm. Online video 4: The video was processed to show the depth details of PdCMs in the adult mouse heart. The video can be found in the following link: https://figshare.com/s/4d9bb0b180cb58c3082f heart size: 7.86 mm × 11.26 mm x 6.113 mm.

Figure 7 shows an identical expression of distribution patterns of the HCN4^+^ cells in Hcn4^DreER/tdTomato^ heart (A) and Pnmt^+^/Tdtomato positive cells in Pnmt^Cre/ChR2−tdTomato^ heart (B).

The results presented here have several advantages and features. First, we used mouse models with conditional cell-type-specific overexpression of fluorescent protein allowing cell-type-specific visualization. These models provide the unique advantage of using tissue clearing for studying specific cell types without the requirement of using immunostaining with antibodies. Second, our modified clearing approach achieves high-quality transparent heart tissue with reduced auto-fluorescent background for optical imaging. Third, imaging by a high-resolution light-sheet system allows global heart view and cellular visualization achieved by rapid changes in optical lens and is integrated with 3D reconstruction without the requirement of additional mathematic modeling. Finally, the 3D reconstruction allows for detailed geometrical and topological analysis. The datasets presented here thus offer a means for reuse and a basis for further development of functional models of the heart by incorporating physiological data in the future.

## Data Availability

The raw data supporting the conclusion of this article will be made available by the authors, without undue reservation.
